# Super-resolution microscopy: revolutionizing life sciences through advanced resolution

**DOI:** 10.52601/bpr.2024.240060

**Published:** 2026-02-28

**Authors:** Shihang Luo, Yan Dong, Lusheng Gu, Wei Ji

**Affiliations:** 1 National Laboratory of Biomacromolecules, Institute of Biophysics, Chinese Academy of Sciences, Beijing 100101, China

## Abstract

With the rapid revolution in super-resolution microscopy, the resolution of far-field optical microscopy has entered the sub-nanometer era, providing new insights into macromolecules *in vitro* and *in situ*.

Super-resolution imaging technology has been recognized by *Nature* as one of the top seven frontier technologies in 2024, underscoring its pivotal role in contemporary scientific research. Since the beginning of the 21^st^ century, this technology has made many groundbreaking advances (Jungmann *et al.* 2014; Rust *et al.* 2006; Willig *et al.* 2006), driven by the deep convergence of physics, chemistry, and life sciences, alongside continuous innovation in both hardware and software. These breakthroughs have profoundly enhanced research across life sciences, particularly in molecular and cellular biology, offering unprecedented opportunities to visualize biological structures at the nanoscale. A notable milestone in this field was the award of the 2014 Nobel Prize in Chemistry to Eric Betzig, Stefan W. Hell, and William E. Moerner, for their pioneering work in super-resolution fluorescence microscopy. Their achievements, which overcame the diffraction limit of traditional optical microscopy, have enabled imaging at nanometer resolution, revolutionizing our ability to explore the molecular intricacies of biological systems.

In recent years, advancements in super-resolution fluorescence microscopy have delivered remarkable progress, with some techniques now pushing spatial resolution to the angstrom (Å) scale. This leap forward provides researchers with powerful tools to investigate biomolecular structures, protein complexes, and intracellular dynamics, with far-reaching implications for the life sciences. The following sections introduce several representative super-resolution imaging techniques in recent years, detailing their underlying principles and exploring their potential applications in biological research.

## MINFLUX

**Figure 1:**
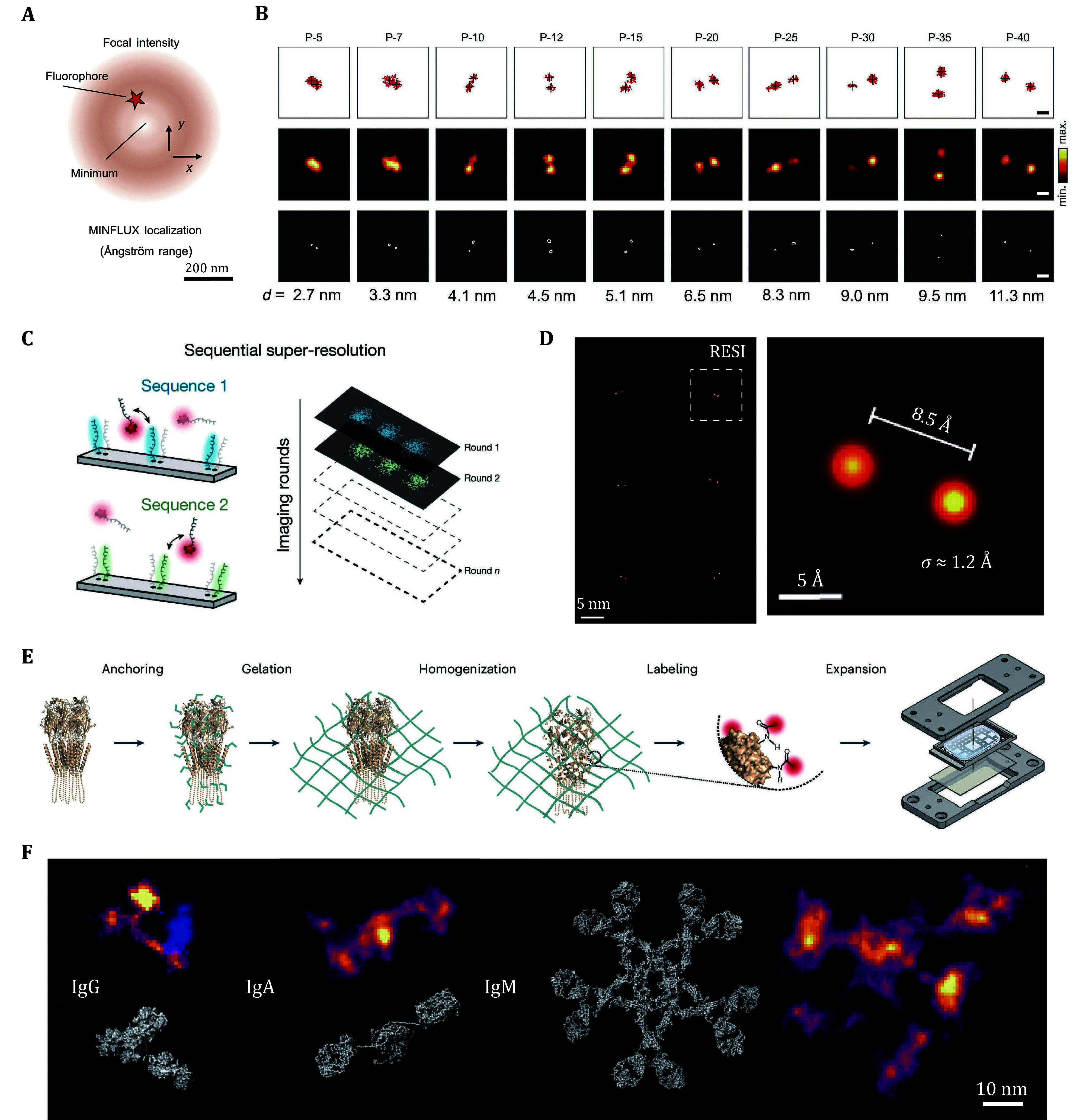
The working principle and resolution of three fluorescence microscopy techniques: MINFLUX, RESI and ONE, (Reinhardt *et al.* 2023; Sahl *et al.* 2024; Shaib *et al.* 2024) respectively. **A** The donut-shaped excitation spot in MINFLUX used to achieve precise localization. **B** MINFLUX reconstructions of polyproline end-to-end distances. **C** RESI achieves sub-nanometer resolution by using orthogonal DNA sequences (blue and green) and sequential acquisition as in Exchange-PAINT. **D** The RESI technique resolves adjacent docking strands and has an average accuracy of 1.2 Å for single base pair backbone distances, with Euclidean distances of 8.5 ± 1.7 Å calculated from individual localizations. **E** The expansion and labeling process of biological samples in ONE method. **F** Imaging of three immunoglobulins (IgG, IgA, IgM) by ONE

In 2024, Steffen J. Sahl and colleagues measured intramolecular spacing as small as 1 nm (as low as 1 Å in planar projection) using MINFLUX fluorescence microscopy (Sahl *et al.* 2024). Previously, Förster resonance energy transfer (FRET) had been utilized for measuring molecular distances and structures in fluorescence microscopy since the 1960s, but the FRET technique is highly nonlinear. The MINFLUX technique uses a "donut" shaped light for excitation (Fig. 1A). The closer the fluorescent molecule is to the center of the ring, the weaker the fluorescence intensity emitted, and the position with the smallest number of photons found through the fit is the precise location of the fluorescent molecule (Balzarotti *et al.* 2017). This study shows that MINFLUX localization determines (internal) molecular distances linearly with sub-nanometer precision at room temperature. In the study, they selected poly-proline chains (poly-proline chain, P-5 to P-40) of different lengths as samples and attached the small molecule fluorescent dye DiMeO-ONB-SiR637 to the ends of the poly-proline chains, and the measurements demonstrated the distribution of the distances of the poly-proline, validating the MINFLUX technology as a "linear measuring scale" (Fig. 1B). Meanwhile, their results also revealed that specific conformations of the dimerization of the PAS domain of histidine kinase, including parallel and antiparallel arrangements, show great potential in cells. Their work shows that fluorescence microscopy is undergoing a seminal shift from merely resolving spatial distributions to precise localization, enabling direct revelation of the biomolecule structure and function with minimal invasiveness.

## RESI

In 2023, Susanne C. M. Reinhardt and colleagues introduced a novel super-resolution microscopy technique, named Resolution Enhancement by Sequential Imaging (RESI) (Reinhardt *et al.* 2023). This technique integrates random labeling with the exchange-PAINT method to achieve angstrom-scale resolution using a conventional fluorescence microscope. In the RESI imaging process, neighboring target molecules are randomly labeled with distinct docking strands. During each imaging round, the imaging strand binds exclusively to the corresponding type of docking strand (Fig. 1C). Once the current imaging round is completed, the imaging strand is replaced with another type for the subsequent round. This sequential imaging strategy ensures that adjacent target molecules correspond to different docking strands in separate imaging cycles, thus emitting fluorescence independently. This approach effectively mitigates the interference between PSFs commonly encountered in traditional DNA-PAINT techniques, allowing angstrom-level resolution to be achieved. The sequential imaging approach of RESI leads to high localization precision. The localization precision in each imaging session is influenced not only by the number of photons detected per localization event (N), but also by the total number of localizations obtained per target (K). By employing a sufficient number of orthogonal labeling sequences, an adequate number of imaging rounds can be performed, resulting in progressively higher resolution. In their work, RESI has achieved the ability to resolve adjacent nucleobases at angstrom resolution (Fig. 1D).

## ONE

In 2024, Susanne C. M. Reinhardt and colleagues introduced a novel imaging technique termed one-step nanoscale expansion microscopy (ONE) (Shaib *et al.* 2024). This method integrates 10x expansion microscopy with the Super-Resolution Radial Fluctuations (SRRF) algorithm (Fig. 1E), achieving enhanced imaging resolution by detecting the intrinsic fluctuations of target fluorescent molecules and accurately localizing them based on the radial symmetry of the sample. Expansion microscopy, in this context, physically enlarges the sample, increasing the distance between labeling sites and thus increasing the effective resolution. This allows the SRRF algorithm to independently resolve the fluctuations of each fluorescent molecule, leading to improved localization precision. The ONE technique has successfully attained 1-nm spatial resolution, enabling the detailed analysis of individual protein structures. They successfully imaged immunoglobulins (IgGs, IgAs and IgMs) and a protein of unknown structure (Fig. 1F). For this protein of unknown structure, its observed results are very similar to the structure predicted for it by AlphaFold. Simultaneously, the researchers visualized the three-dimensional resolution of proteins utilizing the ONE technique. In the case of a human GABAAR homologue, an analysis of 4938 two-dimensional (2D) molecular views facilitated the reconstruction of 3D shapes, which were then compared with structures predicted by AlphaFold and those derived from crystallography. Despite the inherent differences between current fluorescence microscopy and electron microscopy techniques, it remains feasible to derive protein structures from fluorescence imaging data.

In recent years, significant advances have been made in the field of super-resolution fluorescence microscopy, with particular emphasis on the three techniques previously discussed, which represent cutting-edge breakthroughs in this field. These techniques have not only substantially enhanced the spatial resolution of fluorescence microscopy, but in some applications have achieved resolutions approaching those traditionally offered by electron microscopy. Historically, structural biology has relied heavily on electron microscopy techniques, such as cryo-electron microscopy and electron tomography, which are known for their high resolution and ability to resolve complex biomolecular structures. However, with the continuous innovation and refinement of optical super-resolution microscopy techniques, their resolution has now reached the angstrom scale, thus narrowing the gap between fluorescence microscopy and structural biology methodologies. Recent advances in super-resolution fluorescence microscopy are not only due to advancements in hardware, but also to the development and application of various labeling techniques and computational algorithms. For instance, the optimization of fluorescent molecules and the design of novel labeling probes have yielded clearer and more precise molecular signals. Furthermore, sophisticated algorithms, such as SRRF (Salsman and Dellaire 2022), further enhance imaging accuracy and speed by intelligently processing fluorescence signal fluctuations at the molecular level. These developments underscore the transformative impact of computational methods in overcoming traditional optical limitations and enhancing spatial resolution.

Looking ahead, the future of super-resolution fluorescence microscopy appears promising, particularly with the anticipated advancements in hardware, labeling techniques, and the integration of artificial intelligence. Especially, the incorporation of high-performance computing and machine learning algorithms will dramatically increase the speed and accuracy of image data processing (Li *et al.* 2021; Qiao *et al.* 2023; Zelger *et al.* 2018). In parallel, the continued evolution of fluorescent labeling techniques — such as the development of DNA-based nanoprobes and novel dye molecules — will enhance the stability and brightness of fluorescent signals, broadening the applicability of these techniques in complex biological systems. In summary, the ongoing progress in super-resolution fluorescence microscopy holds great potential for driving further innovation in structural biology. As various technologies converge and evolve, fluorescence microscopy is poised to achieve unprecedented levels of resolution, not only in single-molecule imaging and cellular structural analysis, but also in the structural and functional analysis of biological macromolecules. This will provide an invaluable tool for deciphering the fundamental mechanisms underlying life processes, offering novel perspectives that were previously unattainable.
